# INTEREST IN CD2, a global patient-centred study of long-term cervical dystonia treatment with botulinum toxin

**DOI:** 10.1007/s00415-017-8698-2

**Published:** 2017-12-21

**Authors:** Vijay P. Misra, Carlo Colosimo, David Charles, Tae Mo Chung, Pascal Maisonobe, Savary Om, On behalf of the INTEREST IN CD2 study group, A. Abdulnayef, N. U. Adatepe, M. A. Araujo Leite, S. Badarny, O. Bajenaru, M. Bares, P. Bejjani, B. Bergmans, R. Bhidayasiri, H. Bozic, F. E. Cardoso Costa, C. Carlstrom, G. Castelnovo, M. H. Chang, Y. Y. Chang, M. Coletti-Moja, V. Delvaux, P. Dioszhegy, O. Dogu, W. Duzynski, E. Ehler, L. Espinosa Sierra, G. Fabbrini, J. Ferreira, A. Ferreira Valadas, C. Foresti, P. Girlanda, K. J. Goh, A. Graca Velon, S. Grill, T. Gurevitch, M. Hadidi, M. A. Hamimed, A. Hamri, T. Harrower, S. Hassin, P. Hedera, J. F. J. G. Hernandez, J. Hernandez Franco, B. Ho, S. L. Ho, A. Hughes, T. Ilic, J. S. Inshasi, C. W. Ip, S. Jamieson, R. D. G. Jamora, R. Jech, B. S. Jeon, A. Kaminska, M. Karpova, D. Khasanova, J. M. Kim, J. W. Kim, C. Y. Kok, A. Korenko, J. Korv, S. Koussa, T. Kovacs, A. Kreisler, P. Krystkowiak, W. Kumthornthip, C. H. Lin, F. Lundin, G. Lus, M. Magalhaes, A. N. Masmoudi, R. Mercelis, A. Misbahuddin, C. Moebius, B. Mohammadi, B. Nazem, K. Ng, G. Nurlu, J. Nyberg, D. Nyholm, S. Ochudlo, P. Otruba, R. Pfister, Z. Pirtosek, D. Pokhabov, S. Quinones Aguilar, G. Quinones Canales, S. Raghev, H. Rickmann, M. Romano, R. L. Rosales, I. Rubanovits, V. Santilli, L. Schoels, M. Simonetta-Moreau, M. A. Simu, Y. H. Sohn, S. Soulayrol, I. Supe, M. Svetel, T. Sycha, E. K. Tan, S. Timerbaeva, A. B. Tokcaer, R. Trosch, V. Tugnoli, V. Tumas, C. Van der Linden, A. Vetra, C. Vial, E. Vidry, D. Williams, S. Wimalaratna, C. Yiannikas

**Affiliations:** 1 Department of Neurology, Imperial College Healthcare NHS Trust, London, UK; 2 Department of Neurology, Santa Maria University Hospital, Terni, Italy; 3 Vanderbilt Neuroscience Institute, Vanderbilt University Medical Center, Nashville, TN USA; 4 Institute of Physical Medicine and Rehabilitation, São Paulo University Hospital, Sao Paulo, Brazil; 5 Ipsen Pharma, Boulogne-Billancourt, France

**Keywords:** Botulinum toxin, Cervical dystonia, Observational study, Tremor

## Abstract

**Background:**

Longitudinal cohort studies provide important information about the clinical effectiveness of an intervention in the routine clinical setting, and are an opportunity to understand how a population presents for treatment and is managed.

**Methods:**

INTEREST IN CD2 (NCT01753349) is a prospective, international, 3-year, longitudinal, observational study following the course of adult idiopathic cervical dystonia (CD) treated with botulinum neurotoxin type A (BoNT-A). The primary objective is to document long-term patient satisfaction with BoNT-A treatment. Here we report baseline data.

**Results:**

This analysis includes 1036 subjects (67.4% of subjects were female; mean age was 54.7 years old; mean TWSTRS Total score was 31.7). BoNT-A injections were usually given in line with BoNT-A prescribing information. The most commonly injected muscles were splenius capitis (87.3%), sternocleidomastoid (82.6%), trapezius (64.3%), levator scapulae (40.9%) and semispinalis capitis (26.9%); 35.5% of subjects were injected using a guidance technique. Most subjects (87.8%) had been previously treated with BoNT-A (median interval between last pre-study injection and study baseline was 4 months); of these 84.8% reported satisfaction with BoNT-A treatment at peak effect during their previous treatment cycle and 51.5% remained satisfied at the end of the treatment. Analyses by geographical region revealed heterogeneity in the clinical characteristics and BoNT-A injection practice of CD subjects presenting for routine treatment.

**Conclusions:**

These baseline analyses provide sizeable data regarding the epidemiology and clinical presentation of CD, and demonstrate an international heterogeneity of clinical practice. Future longitudinal analyses of the full 3-year study will explore how these factors impact treatment satisfaction.

**Electronic supplementary material:**

The online version of this article (10.1007/s00415-017-8698-2) contains supplementary material, which is available to authorized users.

## Introduction

Cervical dystonia (CD) is the most common form of focal dystonia in adults and is primarily characterised by twisting or turning of the neck causing an abnormal head position [1–3]. Disability with functional impairment, pain, and embarrassment with social withdrawal are also frequent features of CD and several studies have shown that it can have a negative impact on quality of life [4–7]. Treatment guidelines recommend injections of botulinum neurotoxin type A (BoNT-A) as first-line treatment for primary CD [8, 9]. However, there is little data on how BoNT-A injections for CD are administered in routine clinical practice (e.g. dose, duration of effect, choice of muscles to inject, and targeting technique) nor is there robust information on the long-term natural history of CD in patients undergoing treatment.

It is increasingly recognised that while placebo-controlled trials remain the gold standard in assessing response to a therapeutic intervention, they do not provide adequate information of clinical effectiveness and safety of an intervention in the typical clinical setting. In particular, such trials typically employ narrow inclusion and exclusion criteria, and assess only a restricted selection of endpoints. Consequently, they do not provide the answers to many key questions, such as which patients are most likely to benefit from treatment and in what way. Large multicentre longitudinal cohort studies conducted in a routine clinical practice setting are necessary to answer these important questions. Moreover, for generalisability across diverse populations such studies need to have an international geographical representation.

The INTEREST IN CD programme was, therefore, designed to generate real-life data to confirm effectiveness and safety of BoNT-A in routine practice and to create a common language to share knowledge and best practice among physicians and patients. The first study in the programme (INTEREST IN CD1) followed 404 subjects (9 countries) over 1 injection cycle and demonstrated that patient satisfaction is an important and appropriate measure of BoNT-A response in CD subjects [10]. As well as placing greater emphasis on real-world evidence, many regulators and payers now place greater emphasis on patient reported outcomes (PROs) [11, 12], and these organisations often give prominence to the patient voice. Patient satisfaction with treatment is considered especially important as this has been shown to directly correlate with willingness to continue treatment [13, 14]. The INTEREST IN CD2 study was subsequently designed to evaluate the effectiveness of repeated injections of BoNT-A in routine practice. The primary objective of this larger and longer study was to document the effect of BoNT-A treatment upon long-term patient satisfaction with respect to the control of symptoms associated with idiopathic CD.

We report here the baseline characteristics of the INTEREST IN CD2 cohort, which is the largest study in CD to date and was conducted across six continents. While payers have long recognised the need to understand how a clinical population presents for treatment and is managed, clinicians are only starting to understand the relevant insights that real-world studies such as INTEREST IN CD2 can provide [15]. In particular, the scope of the database allowed us to explore the commonalities and differences in international treatment practices, not only in terms of patient presentation, but also injection practice. In addition, the collection of such a standardised and comprehensive dataset also allows interrogation of emerging scientific hypotheses, such as understanding the association between tremor (often a key clinical component of CD) and other CD symptoms.

## Methods

INTEREST IN CD2 (NCT01753349) is an ongoing 3-year multicentre longitudinal cohort study following the course of adult idiopathic CD patients treated with BoNT-A. The study was conducted in compliance with the International Society for Pharmacoepidemiology (ISPE) Guidelines for Good Pharmacoepidemiology Practices (GPP) [16]; it began on 10 December 2012 and last visit of the last patient occurred on 25 September 2017. Participating centres needed to be familiar with Toronto Western Spasmodic Torticollis Rating Scale (TWSTRS) and Tsui scales in their clinical practice. To limit the potential bias that might be introduced by over-recruiting sites, the number of subjects was limited to 12 consecutive subjects per centre. Independent Ethics Committee/Institutional Review Board approval was obtained prior to each centre initiation. Written informed consent was obtained prior to subject enrolment and prior to any data collection.

### Population

This study enrolled adult subjects (≥ legal age in each country) with primary CD presenting for treatment with BoNT-A in routine clinical practice. Subjects could be treated with any BoNT-A formulation. The decision to prescribe a BoNT-A preparation was taken prior to, and independently from, the decision to enrol the subject in the study. Subjects could be new to BoNT-A treatment or previously treated with BoNT-A, provided there had been at least a 12-week interval between the last injection and study entry. Recruitment completed on 31 July 2014, and here we report subjects’ baseline data (baseline visit = Visit 1).

### Assessments

All subjects underwent a comprehensive clinical CD assessment at baseline/first injection visit. An electronic case report form (eCRF) was utilised for data collection, including data on medical history, treatment history, and full details of first injection given (muscles selected, injected dose, injected volume, number of injection sites, use of injection guidance technique). Subjects were also assessed using the TWSTRS [17] and Tsui scale (tremor component) [18].

Subjects previously treated with a BoNT-A reported their satisfaction from last BoNT-A treatment in two ways: (1) their highest level of satisfaction at any time since the last BoNT-A injection and (2) satisfaction at the time of the visit (i.e. baseline visit of the study). Both types of satisfaction were rated on a 5-point Likert scale (1—completely satisfied; 2—rather satisfied; 3—neither satisfied nor dissatisfied; 4—rather dissatisfied; 5—completely dissatisfied).

### Statistical analyses

All analyses of baseline data were made under the guidance of the INTEREST IN CD2 Scientific Committee who regularly met at face-to-face meetings with the Study Team to review trial progress and predefined analyses of interest from the comprehensive dataset. For the purposes of evaluating international differences in CD management, the participating countries were grouped into six regions: Asia, Australia, Europe, Latin America, North Africa/Middle East and the United States of America (USA).

The statistical analyses of this report are primarily descriptive. Mean and standard deviation (mean ± SD) or median measures were used to summarise continuous variables, and absolute and relative frequencies expressed as percentage (%) are presented for categorical information. Analyses of patient satisfaction with BoNT-A treatment were performed in the subgroup of subjects who had previously been treated with BoNT-A. Satisfaction was defined as those with a score of 1 or 2 (completely satisfied or rather satisfied). Associations between Tsui tremor component scores and TWSTRS Total and subscale scores were assessed using a non-parametric Kendall correlation test and then further explored using an analysis of variance.

## Results

A total of 1050 subjects were enrolled from 113 active centres in 34 countries (Online Appendix). Of these, 14 were excluded [data not authenticated (*n* = 7), not injected at Visit 1 (*n* = 4), BoNT-B injected at Visit 1 (*n* = 2), no Visit 1 data available (*n* = 1)] and 1036 subjects were analysed.

### Subject characteristics

The 1036 subjects analysed included 143 from Asia (7 countries), 40 from Australia, 620 from Europe (17 countries), 82 from Latin America (2 countries), 113 from North Africa and the Middle East (6 countries), and 38 from the USA. Subject demographics, medical history, and clinical severity scores at Visit 1 are presented in Table 1. Most subjects were female (67.4% overall), the mean ± SD age was 54.7 ± 13.2 years and the majority of patients had rotation (66.7%) or laterocollis (22.5%) as their predominant CD pattern.

**Table 1:** Demographic, medical history and clinical characteristics at baseline

	Asia (*N* = 143)	Australia (*N* = 40)	Europe (*N* = 620)	Latin America (*N* = 82)	North Africa/ Middle East (*N* = 113)	USA (*N* = 38)	Overall (*N* = 1036)
**Demographics**							
Sex							
Female; *n* (%)	80 (55.9)	32 (80.0)	430 (69.4)	52 (63.4)	71 (62.8)	33 (86.8)	698 (67.4)
Age (years); mean ± SD	55.0 ± 13.5	61.7 ± 12.4	54.7 ± 12.7	56.6 ± 13.6	48.4 ± 13.7	60.3 ± 11.4	54.7 ± 13.2
**Medical history**							
Proportion subjects with CD family history; *n* (%)	3 (2.1)	9 (22.5)	36 (5.8)	5 (6.1)	12 (10.6)	3 (7.9)	68 (6.6)
Time since diagnosis (years); median [range]	6.0 [0.0–29.0]	5.0 [0.0–36.0]	6.0 [0.0–49.0]	7.0 [0.0–57.0]	6.0 [0.0–57.0]	10.0 [0.0–29.0]	6.0 [0.0–57.0]
**Predominant head/neck deviation pattern and associated components** ^a^							
Rotation; *n* (%)	91 (63.6)	31 (77.5)	418 (67.6)	42 (51.2)	75 (66.4)	33 (86.8)	690 (66.7)
Laterocollis; *n* (%)	33 (23.1)	4 (10.0)	146 (23.6)	24 (29.3)	24 (21.2)	2 (5.3)	233 (22.5)
Retrocollis; *n* (%)	9 (6.3)	3 (7.5)	28 (4.5)	12 (14.6)	9 (8.0)	1 (2.6)	62 (6.0)
Anterocollis; *n* (%)	4 (2.8)	1 (2.5)	10 (1.6)	3 (3.7)	2 (1.8)	0 (0)	20 (1.9)
Lateral shift; *n* (%)	4 (2.8)	0 (0)	8 (1.3)	1 (1.2)	1 (0.9)	1 (2.6)	15 (1.5)
Sagittal shift; *n* (%)	2 (1.4)	0 (0)	5 (0.8)	0 (0)	2 (1.8)	1 (2.6)	10 (1.0)
Shoulder elevation; *n* (%)	91 (63.6)	9 (22.5)	263 (42.6)	64 (78.0)	71 (62.8)	19 (50.0)	517 (50.0)
Tremor; *n* (%)	53 (37.1)	29 (72.5)	288 (46.6)	56 (68.3)	55 (48.7)	18 (47.4)	499 (48.3)
Jerk; *n* (%)	12 (8.4)	6 (15.0)	48 (7.8)	20 (24.4)	6 (5.3)	7 (18.4)	99 (9.6)
**Clinical rating scale scores** ^b^							
TWSTRS Total; mean ± SD	32.1 ± 11.9	26.7 ± 12.7	30.5 ± 12.6	39.7 ± 14.2	33.2 ± 14.1	32.5 ± 14.4	31.7 ± 13.1
TWSTRS Severity; mean ± SD	16.9 ± 4.9	13.2 ± 5.1	15.4 ± 5.7	19.2 ± 5.4	16.5 ± 6.0	14.4 ± 5.1	15.9 ± 5.7
TWSTRS Disability; mean ± SD	8.6 ± 6.2	6.7 ± 5.8	9.3 ± 6.1	10.9 ± 7.2	10.5 ± 6.8	11.9 ± 6.6	9.4 ± 6.3
TWSTRS Pain; mean ± SD	6.5 ± 4.9	6.8 ± 5.1	5.8 ± 4.6	9.7 ± 5.3	6.3 ± 4.8	6.2 ± 5.3	6.3 ± 4.9
Tsui tremor score category; *n* (%)							
0	73 (51.0)	8 (20.0)	255 (41.3)	23 (28.0)	50 (44.2)	17 (44.7)	426 (41.2)
1	40 (28.0)	15 (37.5)	183 (29.6)	23 (28.0)	36 (31.9)	9 (23.7)	306 (29.6)
2	18 (12.6)	8 (20.0)	109 (17.6)	26 (31.7)	11 (9.7)	5 (13.2)	177 (17.1)
4	12 (8.4)	9 (22.5)	71 (11.5)	10 (12.2)	16 (14.2)	7 (18.4)	125 (12.1)

Asia: China, Malaysia, Republic of Korea, Singapore, Taiwan, Thailand, The Philippines; Australia; Europe: Austria, Belgium, Czech Republic, Estonia, France, Germany, Hungary, Italy, Latvia, Poland, Portugal, Romania, Russia, Serbia, Slovenia, Sweden, United Kingdom; Latin America: Brazil, Mexico; North Africa and Middle East: Algeria, Israel, Jordan, Lebanon, Turkey, United Arab Emirates; United States of America (USA)

*CD* cervical dystonia, *SD* standard deviation, *TWSTRS* Toronto Western Spasmodic Torticollis Rating Scale, *USA* United States of America

^a^Missing data in two subjects from Europe

^b^Missing data for TWSTRS in one subject from Australia and for Tsui in two subjects from Europe

### Treatment of cervical dystonia

Injection parameters are shown in Table 2. Most subjects were injected at Visit 1 with abobotulinumtoxinA (*n* = 723), followed by onabotulinumtoxinA (*n* = 254) and incobotulinumtoxinA (*n* = 59). Approximately one-third (35.5%) of subjects were injected using a guidance technique, however, there was wide variation between geographical regions. Most guided injections (all regions) were performed using electromyography (EMG, used in 335 of 1036 subjects), while 34 used ultrasound, three electrostimulation and one computed tomography guidance. It was observed that use of injection guidance techniques appeared to be an ‘all or nothing’ practice (i.e. either all muscles or no muscles were injected using a guidance technique).

**Table 2:** Overall BoNT-A injection parameters at baseline

	Asia (*N* = 143)	Australia (*N* = 40)	Europe (*N* = 620)	Latin America (*N* = 82)	North Africa/ Middle East (*N* = 113)	USA (*N* = 38)	Overall (*N* = 1036)
Injected volume (mL); median [range]	2.00 [0.3–8.0]	1.60 [0.3–7.0]	2.00 [0.2–12.5]	2.65 [0.3–10.0]	2.80 [0.2–7.4]	3.00 [1.0–6.0]	2.00 [0.2–12.5]
Number of injection points; median [range]	8.0 [2–24]	5.5 [2–23]	6.0 [1–28]	8.5 [1–34]	8.0 [1–24]	9.0 [2–30]	7.0 [1–34]
Use of injection guidance; *n* (%)	41 (28.7)	37 (92.5)	191 (30.8)	4 (4.9)	72 (63.7)	23 (60.5)	368 (35.5)
BoNT-A dose (U); median [range]							
AbobotulinumtoxinA	*N* = 111 440 [50–800]	*N* = 17 500 [170–1050]	*N* = 428 500 [70–1300]	*N* = 72 500 [50–1700]	*N* = 77 620 [90–1480]	*N* = 18 600 [350–1000]	*N* = 723 500 [50–1700]
IncobotulinumtoxinA	– – –	– – –	*N* = 44 177.5 [50–500]	*N* = 6 245 [170–330]	– – –	*N* = 9 400 [150–500]	*N* = 59 200 [50–500]
OnabotulinumtoxinA	*N* = 32 125 [40–250]	*N* = 23 170 [75–350]	*N* = 148 145 [10–475]	*N* = 4 250 [100–300]	*N* = 36 200 [100–380]	*N* = 11 225 [100–500]	*N* = 254 150 [10–500]

*BoNT-A* botulinum neurotoxin type A, *mL* millilitre, *U* units, *USA* United States of America

More than 17 different muscles were reported as injected by investigators at baseline. The five most commonly injected muscles were the splenius capitis (87.3% of all subjects), sternocleidomastoid (82.6%), trapezius (64.3%), levator scapulae (40.9%) and semispinalis capitis (26.9%). Injection details for those muscles across all regions are given in the Supplementary Appendix. The scalene group (scalenus anterior, posterior and medium) was injected in 16.4% of subjects and other muscles [including the longissimus group (longissimus cervicis, and capitis), oblique capitis group (obliquus capitis inferior, superior and transverse), platysma, splenius cervicis, longus capitis, rectus capitis group (rectus capitis lateralis, posterior major and posterior minor), interspinalis cervicis and longus colli] were injected with a frequency of between 0.2 and 5.0%.

### Satisfaction with previous treatment (previously treated subgroup)

Of the 1036 subjects analysed, 910 were previously treated with BoNT-A and included in this analysis of treatment satisfaction. More subjects had previously been injected with abobotulinumtoxinA compared to onabotulinumtoxinA and incobotulinumtoxinA (64.0 vs. 27.9 and 7.4%, respectively; 0.8% on other BoNT-A). The median time from starting BoNT-A treatment in previously BoNT-treated subjects was 67.1 months [range 2.3–357.0] and the median interval between the last pre-study injection visit and study Visit 1 was 3.6 months [range 1.8–191.5]. The length of treatment interval between the last pre-study injection visit and study Visit 1 differed considerably between regions. For example, whereas most subjects in USA and Australia (81 and 64%, respectively) were re-treated with BoNT-A within 12–16 weeks, most in Latin America and Asia (81 and 73%, respectively) had an injection interval of > 16 weeks. About half in Europe and North Africa/Middle East (52 and 57%, respectively) were treated with an interval of 12–16 weeks and a slightly smaller proportion was treated with an interval of > 16 weeks (45 and 42%, respectively). Less than 3% in all regions were treated with an interval of < 12 weeks. Taken overall, 49% of subjects had an injection interval between last BoNT injection prior to study entry and Visit 1 which was > 16 weeks, 48% between 12 and 16 weeks and 2% < 12 weeks.

**Fig. 1:**
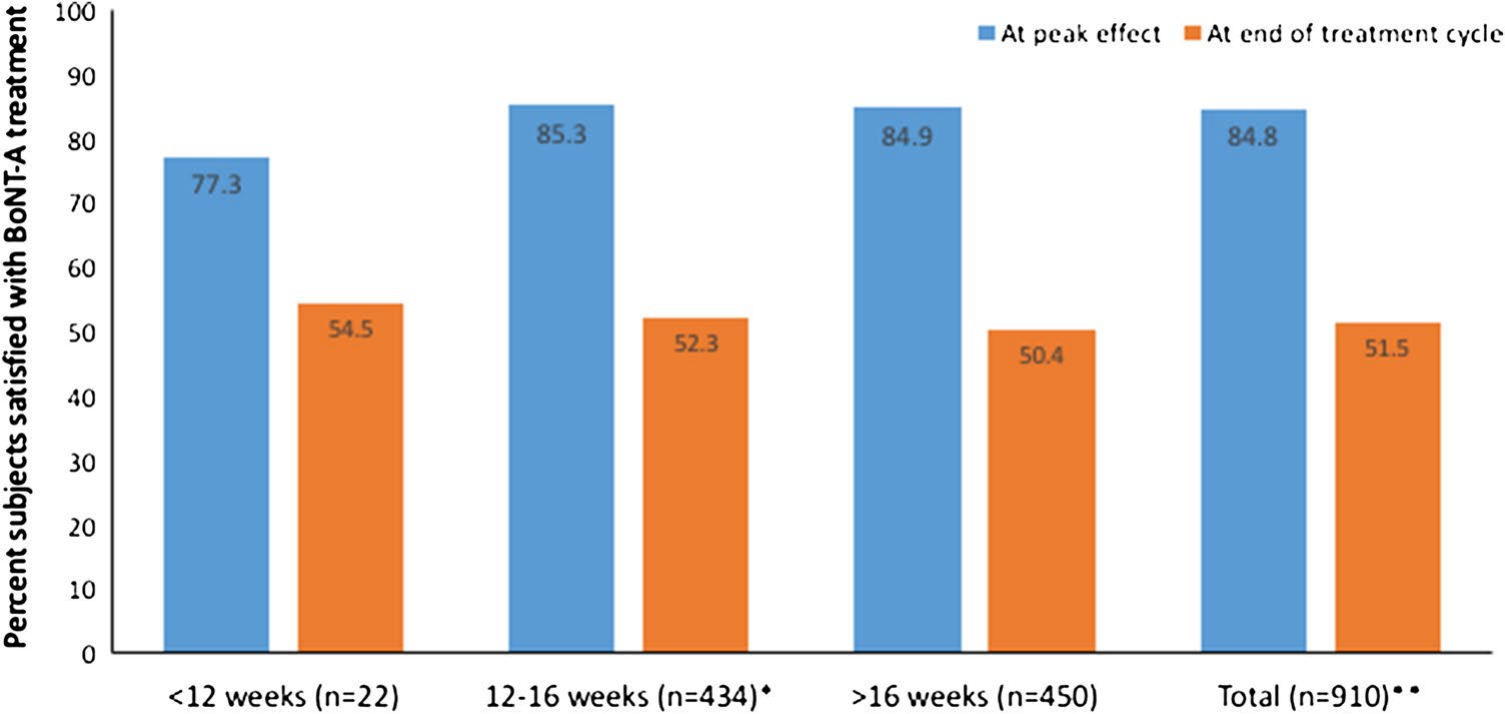
Subject satisfaction with BoNT-A treatment in BoNT previously treated subjects at baseline. *Missing data in one subject from the 12–16 weeks population. **Injection interval between last BoNT injection prior to study entry and baseline visit was unknown in four subjects, however, the satisfaction rating was available for them. *BoNT* botulinum neurotoxin, *BoNT-A* botulinum neurotoxin type A

Figure 1 shows that while 84.8% subjects reported they had been completely/rather satisfied with treatment at peak effect during their previous treatment cycle, fewer (51.5%) reported satisfaction at the end of the treatment cycle (i.e. first study visit). Similar results were seen for all subjects, regardless of the length of the previous treatment interval.

### Association of tremor with CD severity

Non-parametric correlation analysis (based on the total population, *n* = 1036) found a weak association between tremor and TWSTRS Severity. The Kendall’s coefficient was 0.057 with a *p* value of 0.02 (Table 3). Further evaluation using an analysis of variance revealed that subjects with a Tsui score of 4 had significantly higher TWSTRS Total and Severity scores versus subjects with lower Tsui scores (0–2), *P* < 0.01. No other statistically significant associations between Tsui tremor component scores and TWSTRS Total & subscale scores were observed (all correlation coefficients were < 0.1).

**Table 3:** Kendall correlation tests between TWSTRS scores and Tsui tremor scores

Correlation tested	Kendall’s tau-b coefficient	*P* value
TWSTRS Total score and Tsui tremor score	0.040	0.09
TWSTRS Severity score and Tsui tremor score	0.057	0.02
TWSTRS Disability score and Tsui tremor score	0.027	0.27
TWSTRS Pain score and Tsui tremor score	0.019	0.43

*TWSTRS* Toronto Western Spasmodic Torticollis Rating Scale

## Discussion

To the best of our knowledge, INTEREST IN CD2 is the largest study ever conducted in a population of treated CD patients and our data clearly showcase the utility and importance of conducting large multicentre longitudinal cohort studies. In terms of clinical characteristics, the baseline database provides new insights into regional differences in the way patients present for routine treatment for BoNT-A injections. For example, the data showed that subjects in Latin America had the highest TWSTRS Total scores (mean of 39.7), driven by high TWSTRS Pain scores. Likewise, while USA subjects had relatively low TWSTRS Severity scores (mean of 14.4), ratings of TWSTRS Disability were noticeably higher in the USA (mean of 11.9) than all other regions. Such observations may indicate cultural differences in the way CD patients experience their symptoms. However, it is also interesting to note the wide variance in the proportion of females across the regions (from 55.9% representation in Asia to 86.8% representation in the USA) and that subjects recruited from Australia reported a higher proportion of other family members with CD (22.5 vs. < 11% in other regions). While we cannot rule out possible subject selection bias in the different countries and regions, our data highlight a need to further evaluate the impact of regional differences in epidemiological studies investigating age of onset, gender and other differences.

The data generated for injection practice demonstrates that BoNT-A injections are usually given in line with the prescribing information for each product. The predominance in use of abobotulinumtoxinA in this study may reflect a site selection bias, but it should be noted that half (50%) of all sites used more than one product in their patient population. The most commonly injected muscles were appropriate for the most common head/neck deviation patterns (namely, rotational torticollis and laterocollis). While there was commonality across the regions in terms of muscle selection, injection volumes and number of injection sites, there was a striking difference in the use of guidance techniques. This likely reflects the training of injecting clinicians as well as access to equipment. To date, there has been no evidence that use of guidance techniques is required for every patient, and our data appears to support the idea that many injectors feel confident without them. However, we point out that studies have shown that use of a guidance technique improves the safety of injections in patients with complex presentation and with previous experience of adverse events such as dysphagia [19]. Injections of deeper and thinner muscles (such as the levator scapulae and oblique capitis group) often also require injection guidance for accuracy [20].

The INTEREST IN CD1 study [10] demonstrated that patient satisfaction with treatment is a valuable measure of treatment efficacy. Recent surveys have demonstrated that patients have very high expectations for their treatment, with over 60% of patients expecting freedom from spasms and/or freedom from pain and over half expecting to be able to return to a normal routine [7]. As might be expected, overall satisfaction in subjects who had been treated with BoNT for a median of 5.5 years was high, with 84.8% of BoNT previously treated subjects reporting that they were completely or rather satisfied with their previous treatment at peak effect. Satisfaction with treatment on the day of the clinic visit was lower (51.5%) because in most cases at least 12 weeks had passed since the last injection and thus the effects of the last injection were wearing-off or had worn off. Unlike previous studies [21], we observed no differences in the rate of satisfaction (at peak effect or at the time of visit) when subjects were categorised according to the length of their prior treatment interval; patients with an injection interval > 16 weeks appeared to be as equally highly satisfied as those re-injected with a 12–16 week interval. It should be noted that the numbers of subjects with an injection interval of < 12 weeks was too low to draw any conclusions about this short interval. Our assessment of patient satisfaction asked patients to rate their overall ‘control of symptoms’, and did not consider practical ‘health economic’ aspects, such as costs associated with more frequent visits and more frequent injections, that will also affect the patient. For those clinics which must follow a longer than 16-week schedule (e.g. due to reimbursement plans), it may be that a longer lasting toxin formulations would be beneficial—especially as it is clear that satisfaction drops at the end of a treatment cycle.

Updated consensus guidelines for dystonia now emphasise the importance of assessing tremor [22], as an integral feature of this disorder; however, its relationship to other symptoms of CD has not been well studied. Tremor in dystonia usually manifests during posture or voluntary movements, although some dystonic patients may have tremor at rest. Neurophysiological investigations in patients with dystonia (including CD) and tremor show a lack of brainstem interneuronal inhibition, and abnormal sensory integration [23]. Using the unique opportunities provided by the large dataset, we were able to examine whether tremor correlates with other CD symptoms or if it is an independent symptom of CD. In our analyses, the only statistically significant correlation between tremor and CD was a possible association between mean TWSTRS Severity subscore and Tsui tremor severity subscore. However, this association was weak, and the data taken in totality suggest that tremor is an independent symptom of CD (i.e. the severity of tremor does not predict severity of CD).

We believe that the strengths of this study include its size, truly global nature and inclusion of all BoNT-A products. Other studies have been restricted to one product and often one country [24–26]. The study has several limitations including smaller subject numbers in certain regions and site selection bias. Our exploratory ‘regional’ analyses were performed to look for any international heterogeneity of practice; this sometimes required grouping together countries with distinct socio-economic differences to have reasonable sample sizes. Other methods of categorisation could also be of relevance (e.g. by type of healthcare system) and should be analysed in future studies. This is in addition to those limitations inherent to all open-label observational studies. The present report is limited to baseline data, but future data-sets from the study will provide important information about the impact of BoNT-A treatment on the natural history of treated CD (i.e. effectiveness of treatment). Longitudinal subgroup analyses of subjects who were new to BoNT-A treatment will be of particular interest in defining the impact of modern treatment practices on the natural history of CD. We plan to further assess possible predictors of patient satisfaction with treatment, evolution of patient satisfaction after repeated BoNT-A treatment cycles, and how treatment intervals relate to patient satisfaction. One of the key aims of the programme is to share of best practice, and we believe the data presented here will be of practical relevance to BoNT-A injectors.

## Disclosures

This study was sponsored by Ipsen Pharma. Dr VP Misra reports consultancy for Ipsen. Prof C Colosimo reports consultancy for Ipsen, Merz, Zambon, Sunovion and UCB. Vanderbilt University receives income from Abbott, Allergan, Boston Scientific, Ipsen, Lundbeck, Merz, Medtronic, and US WorldMeds for research or educational programmes led by Dr D Charles. Dr D Charles receives income from Allergan, Ipsen, Revance, and the Alliance for Patient Access for education or consulting services. Dr TM Chung reports consultancy for Ipsen. P Maisonobe and Dr S Om are Ipsen employees.

## Electronic supplementary material

Below is the link to the electronic supplementary material.

Supplementary material 1 (PDF 89 kb)
