# Supplementary material for: INTEREST IN CD2, a global patient-centred study of long-term cervical dystonia treatment with botulinum toxin
Source: J Neurol. 2017 Dec 21;265(2):402–9. doi: 10.1007/s00415-017-8698-2 (PMC5808090; doi:10.1007/s00415-017-8698-2)
Supplement: Supplementary file 1 — Supplementary material 1 (PDF 89 kb) [file 415_2017_8698_MOESM1_ESM.pdf]

INTEREST IN CD2, a global patient-centred study of long-term cervical dystonia treatment with botulinum toxin

Vijay P Misra,[1] Carlo Colosimo, David Charles, Tae Mo Chung, Pascal Maisonobe, Savary Om on behalf of the INTEREST IN CD2 study group

1. Imperial College Healthcare NHS Trust, London. peter.misra@virgin.net

Journal of Neurology.

Supplementary Appendix: Injection details for Top 5 most frequently injected muscles at baseline

	Asia (N=143)	Australia (N=40)	Europe (N=620)	Latin America (N=82)	North Africa/ Middle East (N=113)	USA (N=38)	Overall (N=1036)
**Splenius capitis**							
N (%)	113 (79.0)	33 (82.5)	565 (91.1)	68 (82.9)	92 (81.4)	33 (86.8)	904 (87.3)
Injected volume (mL); median [range]	0.8 [0.2-5.2]	0.7 [0.2-3.0]	0.8 [0.1-6.0]	0.8 [0.2-3.5]	0.8 [0.2-2.7]	0.8 [0.3-2.0]	0.8 [0.1-6.0]
Injection points; median [range]	2.0 [1–10]	2.0 [1–9]	2.0 [1–12]	2.0 [1–8]	2.0 [1–6]	2.0 [1–4]	2.0 [1–12]
Injection guidance; n (%)≠	35 (31.0)	32 (97.0)	162 (28.7)	1 (1.5)	65 (70.7)	19 (57.6)	314 (34.7)
BoNT-A dose (U); median [range]							
abobotulinumtoxinA	150 [30-500]	275 [100-500]	200 [15-1000]	150 [50-600]	200 [60-650]	200 [75-300]	200 [15-1000]
incobotulinumtoxinA	-	-	80 [20-185]	40 [30-125]	-	87.5 [25-165]	80 [20-185]
onabotulinumtoxinA	50 [15-130]	75 [18-150]	60 [10-300]	75 [40-100]	60 [25-100]	67.5 [25-200]	60 [10-300]
**Sternocleidomastoid**							
N (%)	125 (87.4)	25 (62.5)	517 (83.4)	72 (87.8)	94 (83.2)	23 (60.5)	856 (82.6)
Injected volume (mL); median [range]	0.63 [0.1-3.2]	0.5 [0.2-1.1]	0.5 [0.1-4.0]	0.75 [0.2-3.0]	0.5 [0.1-2.3]	0.75 [0.2-2.0]	0.5 [0.1-4.0]
Injection points; median [range]	2.0 [1–7]	2.0 [1–5]	2.0 [1–9]	2.5 [1–8]	2.0 [1–8]	3.0 [1–6]	2.0 [1–9]
Injection guidance; n (%)≠	30 (24.0)	23 (92.0)	130 (25.1)	4 (5.6)	62 (66.0)	5 (21.7)	254 (29.7)
BoNT-A dose (U); median [range]							
abobotulinumtoxinA	122.5 [20-350]	150 [100-250]	140 [15-550]	150 [50-400]	100 [50-425]	125 [50-225]	125 [15-550]
incobotulinumtoxinA	-	-	40 [10-120]	55 [40-60]	-	105 [25-200]	50 [10-200]
onabotulinumtoxinA	40 [20-100]	50 [25-75]	40 [10-150]	100 [20-100]	75 [25-113]	50 [15-200]	47.5 [10-200]
**Trapezius**							
N (%)	90 (62.9)	22 (55.0)	392 (63.2)	58 (70.7)	84 (74.3)	20 (52.6)	666 (64.3)

Injected volume (mL); median [range]	0.5 [0.1-2.5]	0.5 [0.2-1.6]	0.6 [0.1-4.5]	0.78 [0.2-5.3]	0.5 [0.1-4.0]	0.88 [0.2-2.0]	0.6 [0.1-5.3]
Injection points; median [range]	2.0 [1–8]	2.0 [1–8]	2.0 [1–12]	4.0 [1–14]	2.0 [1–8]	3.0 [1–6]	2.0 [1–14]
Injection guidance; n (%)≠	24 (26.7)	21 (95.5)	98 (25.0)	4 (6.9)	45 (53.6)	6 (30.0)	198 (29.7)
BoNT-A dose (U); median [range]							
abobotulinumtoxinA	100 [10-320]	100 [100-250]	150 [20-600]	160 [80-1000]	150 [20-1000]	300 [200-400]	150 [10-1000]
incobotulinumtoxinA	-	-	40 [20-115]	50 [20-60]	-	97.5 [25-150]	50 [20-150]
onabotulinumtoxinA	30 [10-80]	40 [25-80]	50 [10-225]	62.5 [20-100]	60 [25-200]	50 [10-100]	50 [10-225]
**Levator scapulae**							
N (%)	49 (34.3)	21 (52.5)	223 (36.0)	42 (51.2)	58 (51.3)	31 (81.6)	424 (40.9)
Injected volume (mL); median [range]	0.35 [0.2-2.5]	0.4 [0.2-2.0]	0.5 [0.1-2.3]	0.5 [0.1-2.4]	0.35 [0.1-1.6]	0.6 [0.3-1.9]	0.4 [0.1-2.5]
Injection points; median [range]	2.0 [1–4]	2.0 [1–3]	1.0 [1–5]	1.0 [1–4]	1.0 [1–3]	1.0 [1–3]	1.0 [1–5]
Injection guidance; n (%)≠	16 (32.7)	19 (90.5)	89 (39.9)	3 (7.1)	40 (69.0)	17 (54.8)	184 (43.4)
BoNT-A dose (U); median [range]							
abobotulinumtoxinA	60 [30-250]	150 [100-400]	100 [25-480]	100 [20-240]	100 [30-200]	150 [75-300]	100 [20-480]
incobotulinumtoxinA	-	-	30 [15-90]	22.5 [10-40]	-	65 [25-100]	40 [10-100]
onabotulinumtoxinA	30 [10-40]	27.5 [10-100]	30 [10-80]	22.5 [20-25]	50 [15-80]	50 [25-100]	30 [10-100]
**Semispinalis capitis**							
N (%)	45 (31.5)	16 (40.0)	147 (23.7)	21 (25.6)	36 (31.9)	14 (36.8)	279 (26.9)
Injected volume (mL); median [range]	0.7 [0.2-2.5]	0.39 [0.2-2.5]	0.5 [0.1-2.0]	0.5 [0.2-1.7]	0.5 [0.1-3.2]	0.4 [0.2-1.0]	0.5 [0.1-3.2]
Injection points; median [range]	2.0 [1–8]	2.0 [1–2]	2.0 [1–8]	2.0 [1–4]	1.5 [1–6]	1.0 [1–4]	2.0 [1–8]
Injection guidance; n (%)≠	11 (24.4)	16 (100.0)	55 (37.4)	0 (0.0)	24 (66.7)	6 (42.9)	112 (40.1)
BoNT-A dose (U); median [range]							
abobotulinumtoxinA	120 [40-350]	125 [100-250]	100 [10-400]	100 [40-340]	100 [30-240]	100 [75-200]	100 [10-400]
incobotulinumtoxinA	-	-	45 [20-90]	25 [20-40]	-	27.5 [15-100]	30 [15-100]
onabotulinumtoxinA	37.5 [10-60]	35 [10-125]	25 [10-70]	-	45 [25-160]	47.5 [45-50]	30 [10-160]

BoNT-A: botulinum neurotoxin type A; mL: millilitre; U: units; USA: United States of America. ≠denominator = number of patients with an injection in that muscle.
